# Transcription Factor RREB1: from Target Genes towards Biological Functions

**DOI:** 10.7150/ijbs.40834

**Published:** 2020-02-21

**Authors:** Ya-Nan Deng, Zijing Xia, Peng Zhang, Samina Ejaz, Shufang Liang

**Affiliations:** 1 State Key Laboratory of Biotherapy and Cancer Center, West China Hospital, Sichuan University and Collaborative Innovation Center for Biotherapy, No.17, 3rd Section of People's South Road, Chengdu, 610041, P.R. China.; 2 Department of Rheumatology, West China Hospital, Sichuan University, Chengdu, 610041, Sichuan, P. R. China.; 3 Department of Urinary Surgery, West China Hospital, West China Medical School, Sichuan University, Chengdu, 610041, P. R. China.; 4 Department of Biochemistry and Biotechnology, Baghdad Campus, The Islamia University of Bahawalpur, Pakistan.

**Keywords:** RREB1, cancer, metabolic disease, MAPK pathway

## Abstract

The Ras-responsive element binding protein 1(RREB1) is a member of zinc finger transcription factors, which is widely involved in biological processes including cell proliferation, transcriptional regulation and DNA damage repair. New findings reveal RREB1 functions as both transcriptional repressors and transcriptional activators for transcriptional regulation of target genes. The activation of RREB1 is regulated by MAPK pathway. We have summarized the target genes of RREB1 and discussed RREB1 roles in the cancer development. In addition, increasing evidences suggest that RREB1 is a potential risk gene for type 2 diabetes and obesity. We also review the current clinical application of RREB1 as a biomarker for melanoma detection. In conclusion, RREB1 is a promising diagnostic biomarker or new drug target for cancers and metabolic diseases.

## Introduction

Zinc finger transcription factors are widely involved in biological processes and play crucial roles in the maintenance of cell activities. RREB1, also known as HNT, FINB and LZ321, is a zinc finger transcription factor that is originally identified as a transcriptional activator of *calcitonin* in response to Ras signaling. The first isoform of RREB1 encodes 755 amino acids and contains four tandem C2H2-type zinc fingers 1. Latter, the finger protein in nuclear bodies (Finb), a longer version of RREB1, is cloned from the cDNA library of breast cancer, which encodes 1656 amino acids with 15 C2H2-type zinc fingers. By now, five RREB1 isoforms RREB1 α, RREB1 β, RREB1 γ, RREB1 δ and RREB1ε are identified.

RREB1 is widely expressed in various human tissues except brain tissue, and it shows relatively higher expression in HeLa and MDA-MB 453 cells among multiple cancer cell lines. RREB1 is mainly located in nuclear body, while its truncated forms have different cell locations. For instance, the truncated variant without 1-974 amino acids locates throughout the nucleus and cytoplasm. While the variant without 1-1407 amino acids only appears in the nucleus and no signal is detected in cytoplasm 2. These evidences suggest a complex mechanism in RREB1 localization. Usually the post-translational modifications (PTMs) are involved in modulation on the activity of transcription factors. The acetylation of RREB1 was reported to be associated with the gene expression, for example HLA-G 3. Moreover, RREB1 is also predicted to be phosphorylated by ERK 1, suggesting that the phosphorylation may play a role in transcription activity of RREB1.

Recently, increasing evidences indicate RREB1 is involved in various biological processes, such as DNA damage repair 4, cell growth and proliferation 5, cell differentiation 1, fat development 6, fasting glucose balance 7, zinc transport 8 and transcriptional regulation. An imbalance of RREB1 function plays a role in the development of various cancers and other diseases including prostate cancer 9, colorectal cancer 5,10, urologic cancer 11, type 2 diabetes 12, leukemia 13 and intervertebral disc degeneration 14. Correspondingly, RREB1 isoforms exhibit different effects on cell growth and proliferation. RREB1β seems to have a more important role than RREB1α in promoting cell growth in UMUC-3 cells 11.

Given that the correlation of RREB1 with the development of various diseases, there is a great possibility to make it a potent disease marker or drug target. The overexpression of RREB1 is correlated with poor survival rate in human Diffuse Large B Cell Lymphoma (DLBCL). In contrast, knockdown of RREB1 inhibits proliferation of DB and Pfeiffer cells 15. RREB1 is also overexpressed in pancreatic cancer compared with normal tissue. Targeting RREB1 expression with RNA interfering *in vitro* and *in vivo* will reduce tumor cell growth of pancreatic cancer 16. RREB1 functions in most cases as a transcription factor to regulate the transcription of target genes. In colorectal cancer, microRNA-143/145 10 and *ITGA7* 5 are the target genes regulated by RREB1. In intervertebral disc degeneration (IDD), RREB1 has been reported to inhibit the expression of ADAMTS5 14. Importantly, these RREB1-targeting genes have been effective drug targets for diseases treatment. These evidences indicate that designing small molecules to target RREB1 and RREB1-regulated genes is feasible for new drug discovery.

In general, more and more evidences show RREB1 is important for cancer occurrence and other diseases. Our review focuses on RREB1-involved signaling pathways, the target genes regulated by RREB1, the role of RREB1 in cancer and disease development and the clinical application of RREB1, which is helpful for comprehensive understanding RREB1 multiple functions in physiological and pathological states.

### RREB1 is a downstream effector of MAPK signaling pathway

Mitogen-activated protein kinase (MAPK) signaling comprises several protein kinases including receptor tyrosine kinases (RTKs), Ras, Raf, MEK and ERK. Aberrant activation of MAPK signaling pathway has been identified in many cancers leading to enhanced survival and metastasis of cancer cells. Among mutations of MAPK signaling pathway, Ras and Raf mutations are the most common ones conferring to cancer cell resistance to chemotherapy or target drug therapy. For example, Kirsten rat sarcoma viral oncogene homolog (KRAS) showed a highly prevalent mutation approximately with 25% of all human cancers.

**Figure 1:**
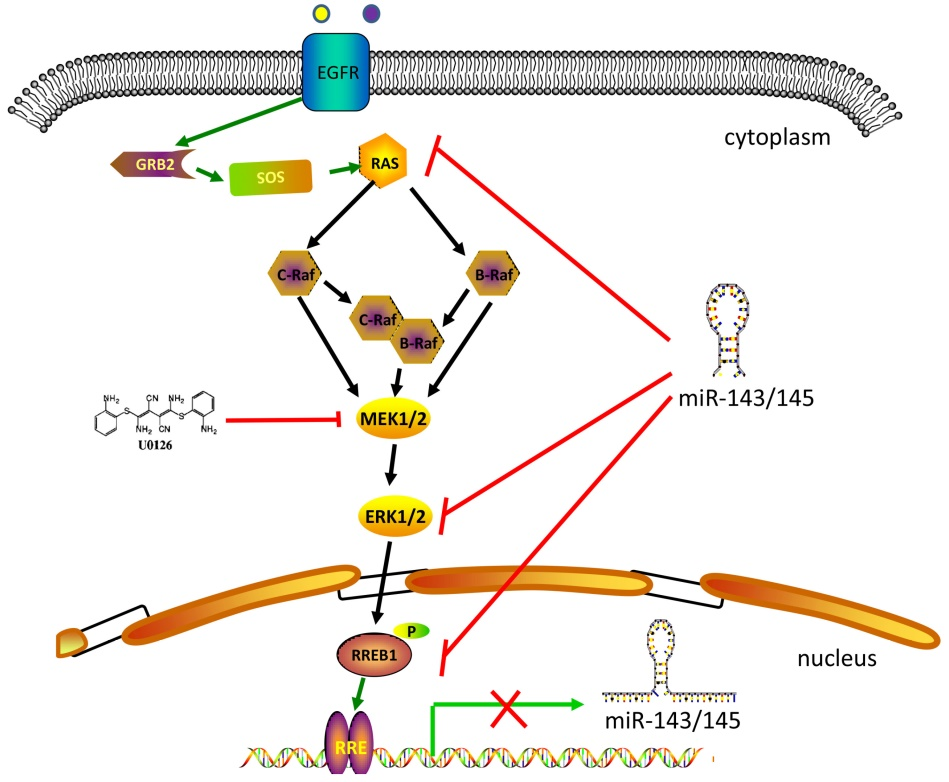
** RREB1-involved in MAPK signaling pathway.** MAPK signaling pathway includes several core members Ras, Raf, MEK1/2 and Erk1/2. Extracellular stimuli activate the MAPK cascade and subsequently the activated Erk1/2 phosphorylates RREB1 to mediate its transcriptional activity. This process will be blocked by MEK1/2 inhibitor U0126. MiR-143 and miR-145 act as tumor suppressor in the pancreatic cancer and colorectal cancer by targeting MAPK signaling pathway. The activated RREB1 binds to RRE in the promoter of miR-143 and miR-145 and inhibits the transcription of miR-143 and miR-145 to remove the inhibition.

RREB1 is confirmed to be a downstream effector of MAPK signaling pathway (Figure 1). The Ras-induced cellular differentiation is characterized by upregulation of the calcitonin (CT) gene. RREB1 binds to the RREs within the promoter of CT gene and activates CT gene. Overexpressing RREB1 increases the expression of CT gene during Ras- or Raf-induced cellular differentiation, which indicates RREB1 is a downstream effector of Ras signaling pathway 1. On the other hand, the activation of RREB1 is dependent on the cascade activation of Ras-Raf-MEK-MAPK pathway. In a transactivation activity test of RREB1/LZ321, transfection of RREB1 alone does not activate the transcription of p321F promoter in H4IIE-C3 cells. In contrast, cotransfection of RREB1 with Raf elevates 4-fold transcription of p321F promoter in H4IIE-C3 cells. This study validates previous finding that Raf activation is required for the activation of RREB1 17. In the Ras-Raf-MEK-MAPK cascade, the activation of MEK is sufficient for the activation of RREB1 18. Another independent study has also supported this hypothesis that RREB1 activation requires phosphorylation downstream of the MAPK pathway. Blocking the MEK activity with U0126 in HPNE-Kras G12D cells induces a decrease of RREB1 mRNA. However, the mRNA level of RREB1 has no change when these cells are treated with AKT inhibitor LY294002. Furthermore, a predicted phosphorylation site within RREB1 has been identified as a substrate of MAPK/ERK1 in two independent groups 1,10.

In addition to being an effector of the Ras signaling pathway, RREB1 is also able to regulate the Ras signaling pathway through repressing miR-143/145 that have been identified as repressor of Ras signaling pathway via targeting genes in Ras pathway including ERK1, ERK5, HGK, JNKK, MEKK, KRAS and RREB1 10. Colorectal cancer cells harboring KRAS or BRAF mutation show a higher expression of RREB1 compared with tissue samples without KRAS or BRAF mutation 10. In addition, RREB1 is also activated by other members of Ras family, such as Ras like proto-oncogene A (RalA) and Ras like proto-oncogene B (RalB) 19.

### Target genes regulated by RREB1

#### RREB1 regulates target genes as a transcriptional activator

RREB1 is also involved in various physiological pathways via activating corresponding genes. RREB1 mediates the secretion regulation of two hormones, secretin and cholecystokinin (CCK). RREB1 interacts with BETA2/NeuroD to promote the transcription of secretin gene 20. Give that lacking an intrinsic domain to independently activate the secretin gene RREB1 exerts its transcriptional promotion through cooperating with other regulatory factors. RREB1 recognizes and directly binds to the Ras-responsive element (RRE) followed by recruitment of DJ-1 to the promoter to fully activate the CCK transcription in a dose-dependent manner 21. Moreover, the expression of *SAMD9L* was regulated by calcitonin. RREB1 also promotes the expression of *SAMD9L,* a Murine paralogue of human* SAMD9,* by binding to the 87 bp sequences upstream of *SAMD9L* transcription start site 22.

In addition, RREB1 is involved in the regulation of insulin production in pancreatic cells. Insulin is responsible for the downregulation of glucose level in blood. NeuroD1 is originally identified as a direct activator of insulin I and insulin II in pancreatic cells 23, 24. RREB1 and NeuroD1 are also identified to occupy the promoter of insulin I and insulin II in pancreatic beta cells, implicating a role of RREB1 in insulin gene regulation. It is noticeable that the epigenetic modification plays a crucial role in RREB1-mediated insulin expression. Mechanistically, RREB1 recruits the H3K9 demethylase LSD1 to remove methyl marks from H3K9Me2. NeuroD1 recruit histone acetyltransferase PCAF to facilitate the acetylation of H3K9 to promote the transcriptional activation of insulin genes.

It is also reported that RREB1 is involved in the regulation of DNA damage response. Under UV condition, RREB1 binds to p53 promotor via interacting with p53 core promoter element (CPE-p53) and promotes the expression of p53. In contrast, silencing RREB1 with RNAi reduces the expression of p53 and p53 target genes, leading to the impairment of p53-meideated protection effect 4.

RREB1 also binds with other gene's regulation region such as the promotor to mediate gene transcription. For instance, Finb/RREB1 is able to bind to the promoter of c-erbB-2, but Finb/RREB1 alone is unable to activate the transcription of c-erbB-2 in COS cells 2. On the contrary, the thymidine kinase (TK) minimal promoter and human metallothionein-IIA (MT-IIA) promoter are efficiently activated by Finb/RREB1. These evidences indicate that RREB1 regulates different target genes in different manners.

#### RREB1 regulates target genes as a transcriptional repressor

A corepressor complex identification by mass spectrometry reveals that RREB1 is a component of CtBP corepressor complex, suggesting that RREB1 may function as a transcriptional repressor 25,26. To date, more than 20 target genes of RREB1 are identified, of which 11 genes are inhibited under different conditions. RREB1 participates in a variety of diseases and biological processes by inhibiting gene expression. RREB1 has been reported to regulate renin-angiotensin system by inhibiting hANG 27 and to participate in the intervertebral disc degeneration protection by inhibiting the expression of ADAMTS5. RREB1 is also involved in the regulation of prostate cancer development by inhibiting hZIP1. Moreover, anti-tumor immunity/immune tolerance was also regulated by RREB1 through targeting HLA-G. In addition, RREB1 participates in the embryonic development regulation by silencing zeta-globin. RREB1 suppresses most of these target genes via a direct binding to the DNA sequence of target gene promoter, except for *PSA* gene. RREB1 does not bind to the promoter of PSA in the absence of androgen receptor (AR) 9.

The binding sequences in the promoter of most of these target genes are in accordance with the pattern of consensus sequence, but each of them is not identical. Moreover, a minor variation in the binding sequence will obviously change the affinity between RREB1 and target sequence. For example, p16^INK4a^ is a known tumor repressor in several cancers by binding to and inhibiting cyclin-dependent kinases 4/6(CDK4/6). The variant of coding region of p16^INK4a^ has been linked to the development of cancers. A recent study indicated that a mutation at regulatory region of p16^INK4a^ is responsible for the development of pristine-induced plasma cell tumors in BALB/c mice. In detail, an 'A' deletion at -32 generates a consensus binding sequence CCCCACACCATCCT and improves the affinity at least four times for RREB1. Luciferase reporter assay suggests that RREB1 plays as a transcription repressor and consequently inhibits the expression of *p16^INK4a^* gene. Decreased expression of p16^INK4a^ results in more susceptible to the pristane-induced plasma cell tumors in BALB/c mice than DBA/2 mice 18.

In addition, RREB1/LZ321 has been reported to bind to GGTCCT that differs from RRE (5'-CCCCACCATCCCC) and consensus binding site (5'-CCCCAAACCACCCC). LZ321 is cloned from human liver cDNA and is identical to RREB1 in the overlapped region. Although RREB1/LZ321 shows a similar affinity with binding to GGTCCT-containing ligand and RRE, GGTCCT-containing element is identified to function as an enhancer. The binding site diversity of RREB1 lays the foundation of target gene diversity and biological function diversity. The role of RREB1 in the zinc uptake is cancer specific. Mentioned above, RREB1 plays a positive role in pancreatic cancer by promoting the expression of ZIP3. However, RREB1 plays a negative role in prostate cancer by binding to the ACCCAAACTTACCC sequence of hZIP1. Mutating ACCCAAACTTACCC into ACCTGAACTTGTCC is sufficient to disrupt the repressive effect of RREB1 on hZIP1 8.

Embryonic zeta-globin genes show a high expression level at early embryonic stage and then are switched off during erythroid development. RREB1 is involved in this transformation via directly binding to the promoter of zeta-globin genes. The increased expression of RREB1 in mature erythroid cell inhibits the expression of zeta-globin genes. Knockdown of RREB1 with RNA interference increases the expression of zeta-globin gene in the human erythroid K562 cell line and primary erythroid culture 13. Therefore, RREB1 is a regulator for zeta-globin gene.

In conclusion, RREB1 functions both transcriptional activator and repressor (Table 1), and its role in target gene regulation may depend on its binding partner and the status of epigenetic modifications. The zinc finger domain allows RREB1 to bind the Ras-responsive element in promoter. Transfecting RREB1 alone is not sufficient to activate or inhibit the expression of some genes. Therefore, uncovering the determinant mechanism of RREB1 transcriptional role is a future direction for researchers.

### The post-translational modifications of RREB1

As we discussed above, RREB1 acts downstream of MAPK signaling pathway and its phosphorylation by ERK may affect its transcriptional activity. Besides phosphorylation, other PTMs, such as acetylation and sumoylation, are widely reported to regulate the activity of transcription factors. The acetylation of RREB1 has been associated with the expression of HLA-G. Acetylated RREB1 will decrease the recruitment of HDAC1 and CtBP1/2 into CtBP repressor complex, which provides a chromatinized environment which allows for recruitment of RNA polymerase II on the transcription start site of *HLA-G* gene in JEG-3 cells. In contrast, reduced acetylation of RREB1 in M8 cells will enhance the interaction with HDAC1 and CtBP1/2 to inhibit transcription of *HLA-G* gene 3.

Sumoylation is also an important modification involved in transcription activity regulation. Analyzing the component of CtBP repressor complex provides another possibility that RREB1 may be modified by sumoylation. Kuppuswamy et al identified ubc9 as a core constituent of the CtBP1 complex, and this complex provides a platform for sumoylation of ZEB1. Similar with ZEB1, RREB1 also functions as a sequence-specific DNA binding repressor in this complex. However, whether RREB1 can be sumoylated through CtBP1 complex is not investigated 28.

### RREB1-mediated activation or inhibition in tumorigenesis

#### Pancreatic cancer

The functional role of RREB1 in pancreatic cancer is still controversial due to multiple mechanisms involvement. RREB1 has been identified a negative regulator of RHO guanine exchange factor ARHGEF2 that is essential for the growth and survival of pancreatic cancer. Relieving the negative regulation of RREB1 on ARHGEF2 contributes to the migratory behavior of pancreatic cancer cells 29. On the other hand, the low level of zinc is crucial to survival of pancreatic cancer cells, and RREB1 inhibits the proliferation of pancreatic cancer through upregulating the zinc level. Meanwhile, the RREB1 transcription factor and ZIP3 zinc uptake transporter were downregulated, leading to the progression of pancreatic cancer. This conclusion is consistent with previous observation that RREB1 is a positive regulator of ZIP3 zinc uptake transporter 30-32.

Studies also propose that RREB1 is an oncogene to promote the phenotype transformation in pancreatic cancer. Bingqing Hui et al 16 reported that RREB1 upregulates the expression of lncRNA AGAP2-AS1 and promotes the proliferation and metastasis in pancreatic cancer through inhibiting the expression of ANKRD1 and ANGPTL4. RREB1 is also involved in the KRAS-induced transformation of pancreatic cancer via repressing the tumor suppressor miR-143/145. In turn, miR-143/145 is capable of repressing RREB1, generating a feed-back mechanism in the KRAS signaling pathway 33. Subsequent studies validated that restoration of miR-143 34 or miR-145 35 retard the transformation of pancreatic cancer at least partly through downregulation of KRAS and RREB1.

#### Prostate cancer

Although there are few reports about RREB1 in prostate cancer, RREB1 is indeed involved in the development of prostate cancer. Nishit K. Mukhopadhyay et al 9 first reported a correlation between RREB1 and prostate cancer. Androgen receptor (AR) is a crucial oncogenic factor in the development of prostate cancer. RREB1 physically interactions with androgen receptor (AR) and binds to the promoter of PSA to inhibit the expression of PSA. This inhibitory effect can be abolished by N-17-Ras or MAPK kinase inhibitor PD98059, which is consistent with the conclusion that RREB1 phosphorylation is MAPK pathway dependent.

Additionally, RREB1 has been reported to downregulate the zinc level in prostate cancer through inhibiting the hZIP1 zinc transporter. Immunohistochemistry with tissue microarrays (TMA) and tissue sections shows an inverse relationship between RREB-1 and hZIP1 8. Expression of RREB1 is significantly increased in prostate cancer tissue. Overexpressed RREB1 decreases the zinc level to provide a microenvironment for the growth of prostate cancer cells. These findings support that RREB1 exerts oncogenic promotion in prostate cancer through different pathways.

#### Colorectal cancer

**Figure 2:**
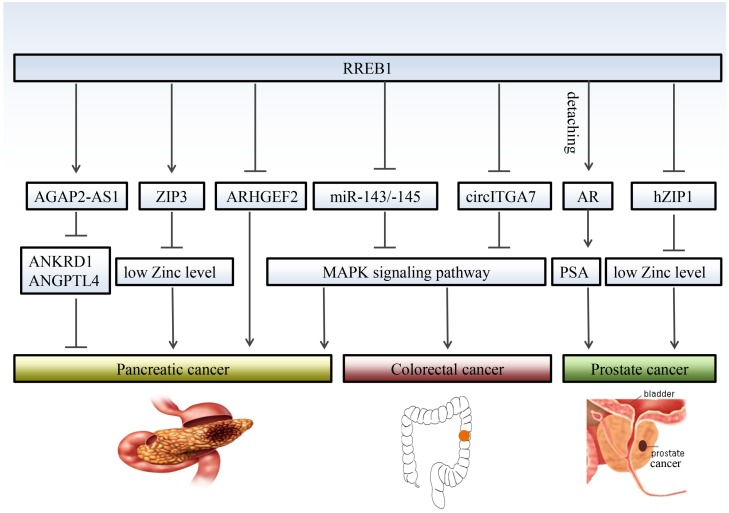
** RREB1 regulates different target genes in different cancers.** RREB1 exhibits multiple mechanisms of action in the development of cancer. Even for the same cancer, RREB1 has different target gene, and different target genes also play different roles in the development of cancer, such as AGAP2-AS1and ZIP3 in prostate cancer. RREB1 promotes the development of colorectal cancer through relieving the inhibition of MAPK pathway by miR-143/145 and circITGA7. Detaching from AR/RREB1 complex, RREB1 restores the expression of PSA.

Colorectal cancer is a common digestive tract malignancy with increasing morbidity in younger people 36. The etiology of colorectal cancer remains largely unknown. RREB1 has been implicated a potential oncogene in colorectal cancer. Oncomine database and immunohistochemistry (IHC) reveal that RREB1 is overexpressed in colorectal cancer tissues compared with normal colon tissues 10. The RREB1 mRNA exhibits 3- to 20-fold higher in colorectal cancer cells harboring active KRAS than that in normal colon tissues. Moreover, the expression of RREB1 shows an inverse relationship with miR-143 and miR-145, two known suppressors of colorectal cancer 37, 38. RREB1 is activated by MAPK pathway and RREB1 activation inhibits the transcription of miR-143/145 by binding to two RREs within its promoter. In turn, overexpression of miR-143/145 represses MAPK pathway through downregulating several targets including KRAS and RREB1. Besides miR-143/145, RREB1 is also regulated by other tumor suppressor, such as a lncRNA circITGA7. It represses the expression of RREB1 via Ras pathway to inhibit growth and metastasis of colorectal cancer. In turn, overexpression of RREB1 will block the inhibitory effect of circITGA7 on Ras pathway by inhibiting ITGA7 to promote colorectal cancer 5 (Figure 2).

#### Melanoma

Frequent amplification of RREB1 in melanoma suggests us the important role of RREB1 in the tumorigenesis of melanoma. As a downstream effector of MAPK signaling pathway, RREB1 inhibits the expression of several tumor suppressors including p53, p16^INK4a^ and miR-143/145 39. Therefore, RREB1 may be a crucial driver-gene to initiate the melanoma by inhibiting tumor suppressors. Another research also supports this hypothesis. Rand et al reported two cases with atypical ALK-positive Spitz tumor in which both exhibited a lack of p16 immunoreactivity and gain of 6p25(RREB1) 40 .

### Pathogenicity of RREB1 in metabolic diseases

#### RREB1 is a potential risk gene for type 2 diabetes

The type 2 diabetes (T2D) is an integrated and multifactorial metabolic disorder that is characterized by insulin resistance and reduced secretion of insulin from pancreatic beta cells 41. Besides age, sex, obesity, low physical activity and a family history of diabetes, many genetic variants contribute to the risk of T2D. With the development of genetic high-resolution technologies, 128 susceptibility genetic markers of T2D have been identified. Genome-wide association studies have demonstrated that some loci associated with the dysregulation of fasting glucose are also the risk alleles for the development of T2D 42,43. In recent several studies, the correlation among RREB1, fasting glucose and T2D has been established. The single nucleotide polymorphisms (SNPs) of RREB1 may be indicators for T2D (Table 2).

Although genetic genes regulating glycemic trait are not necessarily identical to those leading to the conversion to type 2 diabetes, RREB1 is a risk gene responsible for both fasting glucose and T2D. Two SNPs in RREB1 have been linked to the glycemic trait. A recent meta-analysis of GWAS including 133,010 nondiabetic individuals of European ancestry revealed that the rs17762454 in RREB1 is associated with fasting glucose (p<5×10-8), but not with fasting insulin and T2D 7. In another GWAS, rare mutation rs35742417 in RREB1 shows a significant association with fasting glucose 44.

An intronic mutation rs3099797 in RREB1 has been identified as a candidate risk allele for T2D in Starr County Mexican-Americans 45. In another Russian population based investigation, a coding region SNP rs9379084 (p. Asp1171Asn) in RREB1 shows strong association with T2D (p = 0.042) 46. In 2014, a Genome-wide trans-ancestry meta-analyses containing populations from European, East Asian, South Asian, and Mexican and Mexican American ancestry discovered seven novel T2D susceptibility loci, and rs9505118 in RREB1 is one of them (p=1.4×10-9) 12. However, rs9505118 did not achieve a significant level in a subsequent Danish population-based validation study 47. In a subsequent fine-mapping analysis, researchers aggregated coding variant in a larger sample size and they found that the coding variant rs9379084 is the driver factor for the T2D association signal 48, 49. However, another two SNPs of RREB1, rs35742417 (p.Ser1499Tyr) and rs9505118 are excluded. In addition, there are several indirect evidences to support the association of RREB1 with T2D. CDKN2A has been correlated with T2D in GWAS studies. It is reported that the expression of CDNK2A is regulated by RREB1. Furthermore, RREB1 also directly promotes the expression of insulin genes. In conclusion, these evidences suggest that RREB1 shows a strong correlation with T2D, though the exact mechanism remains unclear.

#### RREB1 is a potential risk factor for gestational diabetes mellitus

Gestational diabetes mellitus (GDM) is a complication of pregnancy with increasing prevalence in the world, characterized by glucose intolerance and insulin resistance 50. Similar to T2D, GDM is also influenced by environmental and genetic factors. Moreover, it has been demonstrated that part of susceptibility genes of T2D are casual factors for GDM 51. Two groups have linked T2D risk SNPs rs9505118 and rs9379084 to GDM. Selecting from 45 SNPs of T2D, Kasuga et al has identified three genetic variants including rs9505118 in SSR1-RREB1 as risk SNP of GDM in Japanese population 52. Another case-control study containing 2636 women with GDM and 6086 non-GDM control women identified eight genetic variants that were significantly associated with GDM, and rs9379084 in RREB1 was the one of eight genetic variants. However, a recent GDM study in Asian Indian population showed that RREB1 is excluded from the list of risk genes at least in this population, which suggests that different population may harbor their own specific risk genes for GDM 53.

Understanding the underlying mechanism of GDM is important for the health of offspring of mothers with GDM. It has been reported that the mothers with GDM increased the risk of developing T2D in their offspring 54. Exposure to hyperglycemia during pregnancy may induce changes in DNA methylation 55. A recent study of siblings revealed that intrauterine exposure to maternal gestational diabetes will significantly increase the methylation of RREB1 in offspring compared to non-GDM groups 56. Although there is no experimental evidence to elucidate the exact role of RREB1 in the development of GDM, we need to note that RREB1 has been associated with fasting glucose and T2D.

#### RREB1 is involved in fat development and distribution

Recent GWAS studies identified RREB1 as a candidate gene for fat development and distribution. A report demonstrates that rs6931262 at RREB1 has a significant association with body fat distribution in African ancestry population (p=2.48×10^-8^) 57. In a multiehnic meta-analysis of up to 18332 participants from European, African, Hispanic and Chinese ancestry, another variation rs2842895 in RREB1 is associated with visceral adipose tissue(VAT) (p = 1.1 × 10^-8^), but not with pericardial adipose tissue (PAT) and subcutaneous adipose tissue (SAT). However, subsequent functional studies demonstrate that RREB1 is not differentially expressed in these three tissues and do not differ in response to the obesogenic stimulus and in adipogenic induction 6.

In addition, RREB1 is indeed involved in the brown fat (BAT) development. RREB1 selectively binds to H3K27me3 marked promoter of BAT-selective genes, such as Ucp1 and Cidea, and recruits Jmjd3 to remove the H3K27me3, leading to the expression of BAT-selective genes. Furthermore, the expression of RREB1 is much higher in brown fat tissues and white fat tissues. RREB1 is strongly elevated during brown adipogenesis, but knockdown of RREB1 has no effect on brown adipogenesis 58. In total, RREB1 and RREB1-meidated histone modification play an important role in fat development and distribution.

#### RREB1 is a potential drug target for diseases treatment

RREB1 is widely involved in the development of tumorigenesis and metabolic diseases by regulating various target genes. Suppression of RREB1 with inhibitors will reverse the effects of target genes on the diseases progression. Although no small molecule compounds targeting RREB1 have been reported by now, there are many studies on RREB1 target genes for drug development. In pancreatic and colorectal cancer, RREB1 overexpression will promote cancer cell proliferation through the inhibition of tumor suppressor miR-143/145. Actually, restore of miR-143/145 will reduce tumor growth. In fact, using lipid-based nanoparticle (nanovector) to deliver the tumor suppressor "TSG-miRs" miR-34a or miR-143/145 to MiaPaCa-2 indeed inhibits the pancreatic tumor growth 59.

MiR-143/145 replacement therapy was also reported in colorectal cancer. Chemical-modified miR-143 improved its activity and stability, leading to a significant suppressive effect on colorectal cancer in vitro and in vivo 60. Even delivering unmodified miR-145 with polyethylenimine (PEI) will reach the comparable suppressive effect on colorectal cancer 61. On the other hand, RREB1 inhibition by circITGA7 will increase the expression of ITGA7 to inhibit colorectal cancer growth 5. Therefore, targeting RREB1 with small molecule compounds will be a promising way for cancer therapy.

### Diagnostic value of RREB1 in melanoma detection

Because RREB1 plays an important role in tumorigenesis, RREB1 is a good marker for tumor diagnosis, prognosis prediction and management of patients. At present, the clinical application of RREB1 is mainly used as a molecular diagnostic marker for melanoma.

Accurate classification of melanocytic tumors as benign or malignant is crucial for patient diagnosis and treatment. The routine method for melanoma detection is histopathological detection. However, it is hard to distinguish melanocytic tumors sharing common features of nevi and melanoma through conventional morphological features analysis. Previous studies have revealed that chromosomal aberrations frequently occurred in melanomas, but not in benign nevi 62, 63. Comparative genomic hybridization (CGH) demonstrates that melanoma harbors copy number gains of 1q, 4q, 6p, 7q, 8q, 11q, 17q and 20q, and frequent loss of 9p,10p,10q , 21q 64 and 6p 65. A subsequent study based on a training set of 301 tumors generates a panel of 6p25 (*RREB1*), 6q23 (*MYB*), Cep6, and 11q13 (*CCND1*) to distinguish melanoma and nevi, and this panel shows a sensitivity of 86.7% and specificity of 95.4% in the final validation sets 66. The usefulness of this panel is confirmed by other studies in different types of melanomas. For example, MOREY et al validated this panel in the diagnosis of cutaneous melanocytic tumors, reaching a sensitivity of 90% and a specificity of 95% 67. Another case in distinguishing BN-like cutaneous melanoma metastasis from conventional blue nevi or epithelioid blue nevi (EBN) also shows high sensitivity and specificity 68. In addition, the panel of 6p25 (*RREB1*), 6q23 (*MYB*), Cep6, and 11q13 (*CCND1*) do work in distinguishing nevoid melanoma from mitotically active nevi 69. However, the diagnostic capability of this panel varies greatly in different subtypes of melanomas. This panel is the most sensitive in the subgroups of nodular and acral melanomas and is the least sensitive in the superficial spreading subtype. It is of note that the role of *RREB1* in melanoma diagnosis is particularly important. 6p25(*RREB1*) showed the most sensitive diagnosis of melanoma in overall cases as well as in each of different subtypes of melanomas, which revealed a strong correlation with melanomas. A higher sensitivity of gain in 6p25 (*RREB1*) compared to CEP6-related *MYB* loss, *CCND1* gain and *MYB* gain was also observed in an evaluation of 50 melanocytic skin lesions 70.This is supported by another report. The patients with the gain of 6p where RREB1 locates in show a lower survival rate (33.3%) than those without the gain of 6p (92.9%), indicating that the gain of 6p may be a poor prognostic indicator 65.

Despite the sensitivity of 4-color FISH in distinguishing melanoma from nevi, it is difficult to diagnose Spitz nevi due to its significant overlap with melanomas 71.To further improve the accuracy of diagnosis in morphologically ambiguous melanocytic neoplasms and Spitzoid neoplasms, Gerami et al proposes a new probe panel including 9p21 (*CDKN2a*), 6p25 (*RREB1*), 11q13 (*CCND1*) and 8q24 (*MYC*). The new probe set shows a higher sensitivity of 94% and specificity of 98% compared with the previous probe set that showed a sensitivity of 75% and specificity of 96% in the same validation data set, which improves the discriminatory power in distinguishing melanoma from nevi. They finally proposed a new solution that adding 9p21 (*CDKN2a*) into previous 4-probe FISH assay for spitzoid melanomas diagnosis. However, few studies are conducted with new probe set. Moreover, it is also a challenge for borderline melanocytic tumor (BMT) diagnosis 72.

### Conclusion and perspective

As an effector of MAPK pathway, RREB1 is involved in cell growth, cell differentiation and DNA damage repair. RREB1 functions as both a transcriptional activator and a transcriptional repressor. The transcriptional activity of RREB1 is also dependent on its post-transcriptional modification, such as phosphorylation. In addition, acetylation may also be involved in the regulation of RREB1 activity. Analysis of the transcriptional repressor complex revealed that RREB1 can act together with deacetylase or demethylase to regulate transcription. It has been found that the interaction of RREB1 with different genes or different modifications of RREB1 may affect its transcriptional function, but the specific mechanism of action is still not fully explored.

Though RREB1 exerts an oncogene or repressor gene in several cancers, the role of RREB1 in pancreatic cancer is still controversial. The indirect evidences indicate that RREB1-mediated immune mechanism may be involved in the regulation of cancer development. Considering a feedback among RREB1, miR-143/145 and KRAS, Zhou et al proposed a miR-143-meidated immune evasion in colorectal cancer. TGF-β 1 will elevate the expression of miR-155, and miR-155 decreases the expression of miR-143 by inhibiting CEBPB. Subsequently, the expression of B7-H3 and B7-H4 are augmented due to the release of miR-143 inhibitory effect. Overexpressed B7-H3 and B7-H4 induce T cells to secret TGF-β 1 and immunosuppressive cytokines IL-2, IL-6 and IL-17. Despite B7-H3 and B7-H4, other coinhibitory factor B7-DC, CTLA4 and PD-1 are also inhibited by miR-143 73. In addition, KRAS-mediated KAP1/TRIM28 sumoylation is also involved in the KRAS-driven transformation in colorectal cancer 74. The role of KAP1/TRIM28 in immunomodulatory has been widely reported 75,76. However, whether RREB1-mediated immunomodulatory is involved in cancer development is still to be investigated.

RREB1 also plays an important role in the development of metabolic diseases, such as diabetes. Multiple SNPs in RREB1 are associated with the risk of developing diabetes. Therefore, RREB1 may also be one of the pathogenic genes of diabetes. And this is supported by recent new findings that insulin gene is a target of RREB1. RREB1 is associated with the regulation of fasting glucose. RREB1 also regulates the distribution and synthesis of fat. These evidences suggest that RREB1 plays an important role in diabetes, but the specific mechanism remains unclear.

The importance of targeting RREB1 or downstream target genes is significant. The ectopic expression of RREB1 in diseases has been widely reported. Moreover, RREB1 always exerts its oncogenic role through regulating the expression of downstream target genes. Therefore, blocking RREB1 or its target genes is an efficient way for diseases treatment. In the future, with the deep research on RREB1, it will be possible to provide new potential therapeutic targets for cancers and metabolic diseases.

## Figures and Tables

**Table 1:** Target gene of RREB1 in cancers and diseases

Potential role	Target gene	Cancer or disease	Binding sequence	Ref
activator	Calcitonin	human medullary thyroid cancer cell line TT	CCCCAAACCACCCC	1
activator	TK,			2
	MT-IIA,			
	TTK			17
activator	P53		AAAACCCCAATCCCATCAACCCCTC	4
activator	Secretin,	intestinal and pancreatic endocrine cells		20
	ß-glucokinase,			
	insulin I,			
	insulin II			
activator	AGAP2-AS1	pancreatic cancer		16
activator	ZIP3	pancreatic cancer		32
activator	SAMD9L			22
activator	CCK		CCCCAACCCCCCCA	21
activator	Ucp1	brown adipocytes		58
	Cidea			
repressor	p16 ^INK4a^	BAL B/C mice	CCCCACACCATCCT	18
repressor	hANG		GGATGG-like	27
repressor	PSA	prostate cancer		9
repressor	HLA-G		GGTCCT	3
repressor	zeta -Globin Gene	erythroid cell lines		13
repressor	miR-143/145	pancreatic cancers And colorectal cancer		10,33
repressor	hZIP1	Prostate cancer		8
repressor	ARHGEF2	pancreatic cancer		29
repressor	ADAMTS5	nuclear pulposus(NP) cells		14
repressor	ITGA7	Colorectal cancer		5
repressor	PTPRG	childhood acute lymphoblastic leukemias		77

**Table 2:** SNPs of RREB1 which are associated with type 2 diabetes and fasting glucose

SNP	Amino acid alteration	Associated phenotypes	….population	Ref
rs3099797	intron_variant	T2D	Mexican-Americans	45
rs9379084	p.Asp1171Asn	T2D	Russian	46
rs9505118	intron_variant	T2D	European, East Asian, South Asian, and Mexican and Mexican American ancestry	12
rs9505118	intron_variant	T2D (not significant)	Danish	47
rs35742417	p.Ser1499Tyr	fasting glucose	non-diabetic individuals of European ancestry	44
rs17762454	intron_variant	fasting glucose	European ancestry without diabetes	7

## References

[1] Thiagalingam A, De Bustros A, Borges M, Jasti R, Compton D, Diamond L (1996). RREB-1, a novel zinc finger protein, is involved in the differentiation response to Ras in human medullary thyroid carcinomas. Mol Cell Biol.

[2] Fujimoto-Nishiyama A, Ishii S, Matsuda S, Inoue J, Yamamoto T (1997). A novel zinc finger protein, Finb, is a transcriptional activator and localized in nuclear bodies. Gene.

[3] Flajollet S, Poras I, Carosella ED, Moreau P (2009). RREB-1 is a transcriptional repressor of HLA-G. J Immunol.

[4] Liu H, Hew HC, Lu ZG, Yamaguchi T, Miki Y, Yoshida K (2009). DNA damage signalling recruits RREB-1 to the p53 tumour suppressor promoter. Biochem J.

[5] Li X, Wang J, Zhang C, Lin C, Zhang J, Zhang W (2018). Circular RNA circITGA7 inhibits colorectal cancer growth and metastasis by modulating the Ras pathway and upregulating transcription of its host gene ITGA7. J Pathol.

[6] Chu AY, Deng X, Fisher VA, Drong A, Zhang Y, Feitosa MF (2017). Multiethnic genome-wide meta-analysis of ectopic fat depots identifies loci associated with adipocyte development and differentiation. Nat Genet.

[7] Scott RA, Lagou V, Welch RP, Wheeler E, Montasser ME, Luan JA (2012). Large-scale association analyses identify new loci influencing glycemic traits and provide insight into the underlying biological pathways. Nat Genet.

[8] Zou J, Milon BC, Desouki MM, Costello LC, Franklin RB (2011). hZIP1 Zinc transporter down-regulation in prostate cancer involves the overexpression of Ras responsive element binding protein-1 (RREB-1). Prostate.

[9] Mukhopadhyay NK, Cinar B, Mukhopadhyay L, Lutchman M, Ferdinand AS, Kim J (2007). The zinc finger protein ras-responsive element binding protein-1 is a coregulator of the androgen receptor: implications for the role of the ras pathway in enhancing androgenic signaling in prostate cancer. Mol Endocrinol.

[10] Kent OA, Fox-Talbot K, Halusha MK (2013). RREB1 repressed miR-143/145 modulates KRAS signaling through downregulation of multiple targets. Oncogene.

[11] Nitz MD, Harding MA, Smith SC, Thomas S, Theodorescu D (2011). RREB1 transcription factor splice variants in urologic cancer. Am J Pathol.

[12] Mahajan A, Go MJ, Zhang WH, Below JE, Gaulton KJ, Ferreira T (2014). Genome-wide trans-ancestry meta-analysis provides insight into the genetic architecture of type 2 diabetes susceptibility. Nat Genet.

[13] Chen RL, Chou YC, Lan YJ, Huang TS, Shen CKJ (2010). Developmental silencing of human zeta-globin gene expression is mediated by the transcriptional repressor RREB1. Journal of Biological Chemistry.

[14] Wang K, Song Y, Liu W, Wu XH, Zhang YK, Li SH (2017). The noncoding RNA linc-ADAMTS5 cooperates with RREB1 to protect from intervertebral disc degeneration through inhibiting ADAMTS5 expression. Clin Sci.

[15] Rahrmann EP, Wolf NK, Otto GM, Heltemes-Harris L, Ramsey LB, Shu JM (2019). Sleeping Beauty Screen Identifies RREB1 and Other Genetic Drivers in Human B-cell Lymphoma. Molecular Cancer Research.

[16] Hui B, Ji H, Xu Y, Wang J, Ma Z, Zhang C (2019). RREB1-induced upregulation of the lncRNA AGAP2-AS1 regulates the proliferation and migration of pancreatic cancer partly through suppressing ANKRD1 and ANGPTL4. Cell Death Dis.

[17] Zhang L, Zhao JH, Edenberg HJ (1999). A human Raf-responsive zinc-finger protein that binds to divergent sequences. Nucleic Acids Res.

[18] Zhang S, Qian X, Redman C, Bliskovski V, Ramsay ES, Lowy DR (2003). p16 INK4a gene promoter variation and differential binding of a repressor, the ras-responsive zinc-finger transcription factor, RREB. Oncogene.

[19] Oxford G, Smith SC, Hampton G, Theodorescu D (2007). Expression profiling of Ral-depleted bladder cancer cells identifies RREB-1 as a novel transcriptional Ral effector. Oncogene.

[20] Ray SK, Nishitani J, Petry MW, Fessing MY, Leiter AB (2003). Novel transcriptional potentiation of BETA2/NeuroD on the secretin gene promoter by the DNA-binding protein Finb/RREB-1. Mol Cell Biol.

[21] Yamane T, Suzui S, Kitaura H, Takahashi-Niki K, Iguchi-Ariga SM, Ariga H (2013). Transcriptional activation of the cholecystokinin gene by DJ-1 through interaction of DJ-1 with RREB1 and the effect of DJ-1 on the cholecystokinin level in mice. PLoS One.

[22] Jiang QJ, Quaynor B, Sun A, Li QL, Matsui H, Honda H (2011). The samd9L gene: transcriptional regulation and tissue-specific expression in mouse development. J Invest Dermatol.

[23] Naya FJ, Stellrecht CM, Tsai MJ (1995). Tissue-specific regulation of the insulin gene by a novel basic helix-loop-helix transcription factor. Genes Dev.

[24] Qiu Y, Sharma A, Stein R (1998). p300 mediates transcriptional stimulation by the basic helix-loop-helix activators of the insulin gene. Mol Cell Biol.

[25] Shi YJ, Matson C, Lan F, Iwase S, Baba T, Shi Y (2005). Regulation of LSD1 histone demethylase activity by its associated factors. Mol Cell.

[26] Shi Y, Sawada J, Sui G, Affar el B, Whetstine JR, Lan F (2003). Coordinated histone modifications mediated by a CtBP co-repressor complex. Nature.

[27] Date S, Nibu Y, Yanai K, Hirata J, Yagami K, Fukamizu A (2004). Finb, a multiple zinc finger protein, represses transcription of the human angiotensinogen gene. Int J Mol Med.

[28] Kuppuswamy M, Vijayalingam S, Zhao LJ, Zhou Y, Subramanian T, Ryerse J (2008). Role of the PLDLS-binding cleft region of CtBP1 in recruitment of core and auxiliary components of the corepressor complex. Mol Cell Biol.

[29] Kent OA, Sandi MJ, Burston HE, Brown KR, Rottapel R (2017). An oncogenic KRAS transcription program activates the RHOGEF ARHGEF2 to mediate transformed phenotypes in pancreatic cancer. Oncotarget.

[30] Costello LC, Zou J, Desouki MM, Franklin RB (2012). Evidence for changes in RREB-1, ZIP3, and Zinc in the early development of pancreatic adenocarcinoma. J Gastrointest Cancer.

[31] Franklin RB, Zou J, Costello LC (2014). The cytotoxic role of RREB1, ZIP3 zinc transporter, and zinc in human pancreatic adenocarcinoma. Cancer Biol Ther.

[32] Costello LC, Levy BA, Desouki MM, Zou J, Bagasra O, Johnson LA (2011). Decreased zinc and downregulation of ZIP3 zinc uptake transporter in the development of pancreatic adenocarcinoma. Cancer Biol Ther.

[33] Kent OA, Chivukula RR, Mullendore M, Wentzel EA, Feldmann G, Lee KH (2010). Repression of the miR-143/145 cluster by oncogenic Ras initiates a tumor-promoting feed-forward pathway. Gene Dev.

[34] Pham H, Rodriguez CE, Donald GW, Hertzer KM, Jung XS, Chang HH (2013). miR-143 decreases COX-2 mRNA stability and expression in pancreatic cancer cells. Biochem Biophys Res Commun.

[35] Sureban SM, May R, Qu D, Weygant N, Chandrakesan P, Ali N (2013). DCLK1 regulates pluripotency and angiogenic factors via microRNA-dependent mechanisms in pancreatic cancer. PLoS One.

[36] Weinberg BA, Marshall JL, Salem ME (2017). The growing challenge of young adults with colorectal cancer. Oncology (Williston Park).

[37] Slaby O, Svoboda M, Fabian P, Smerdova T, Knoflickova D, Bednarikova M (2007). Altered expression of miR-21, miR-31, miR-143 and miR-145 is related to clinicopathologic features of colorectal cancer. Oncology-Basel.

[38] Michael MZ, SM OC, van Holst Pellekaan NG, Young GP, James RJ (2003). Reduced accumulation of specific microRNAs in colorectal neoplasia. Mol Cancer Res.

[39] Turri-Zanoni M, Medicina D, Lombardi D, Ungari M, Balzarini P, Rossini C (2013). Sinonasal mucosal melanoma: Molecular profile and therapeutic implications from a series of 32 cases. Head Neck-J Sci Spec.

[40] Rand AJ, Flejter WL, Dowling CA, Brooke LM, Boland GM, Kroshinsky D (2018). Atypical ALK-positive Spitz tumors with 9p21 homozygous deletion: Report of two cases and review of the literature. J Cutan Pathol.

[41] Hameed I, Masoodi SR, Mir SA, Nabi M, Ghazanfar K, Ganai BA (2015). Type 2 diabetes mellitus: From a metabolic disorder to an inflammatory condition. World J Diabetes.

[42] Dupuis J, Langenberg C, Prokopenko I, Saxena R, Soranzo N, Jackson AU (2010). New genetic loci implicated in fasting glucose homeostasis and their impact on type 2 diabetes risk. Nat Genet.

[43] Voight BF, Scott LJ, Steinthorsdottir V, Morris AP, Dina C, Welch RP (2010). Twelve type 2 diabetes susceptibility loci identified through large-scale association analysis. Nat Genet.

[44] Mahajan A, Sim X, Ng HJ, Manning A, Rivas MA, Highland HM (2015). Identification and functional characterization of G6PC2 coding variants influencing glycemic traits define an effector transcript at the G6PC2-ABCB11 locus. Plos Genet.

[45] Below JE, Gamazon ER, Morrison JV, Konkashbaev A, Pluzhnikov A, McKeigue PM (2011). Genome-wide association and meta-analysis in populations from Starr County, Texas, and Mexico City identify type 2 diabetes susceptibility loci and enrichment for expression quantitative trait loci in top signals. Diabetologia.

[46] Barbitoff YA, Serebryakova EA, Nasykhova YA, Predeus AV, Polev DE, Shuvalova AR (2018). Identification of novel candidate markers of type 2 diabetes and obesity in russia by exome sequencing with a limited sample size. Genes-Basel.

[47] Harder MN, Appel EVR, Grarup N, Gjesing AP, Ahluwalia TS, Jorgensen T (2015). The type 2 diabetes risk allele of TMEM154-rs6813195 associates with decreased beta cell function in a study of 6,486 danes. Plos One.

[48] Fuchsberger C, Flannick J, Teslovich TM, Mahajan A, Agarwala V, Gaulton KJ (2016). The genetic architecture of type 2 diabetes. Nature.

[49] Mahajan A, Wessel J, Willems SM, Zhao W, Robertson NR, Chu AY (2018). Refining the accuracy of validated target identification through coding variant fine-mapping in type 2 diabetes. Nat Genet.

[50] Zhu YY, Zhang CL (2016). Prevalence of gestational diabetes and risk of progression to type 2 diabetes: a global perspective. Curr Diabetes Rep.

[51] Lauenborg J, Grarup N, Damm P, Borch-Johnsen K, Jorgensen T, Pedersen O (2009). Common type 2 diabetes risk gene variants associate with gestational diabetes. J Clin Endocrinol Metab.

[52] Kasuga Y, Hata K, Tajima A, Ochiai D, Saisho Y, Matsumoto T (2017). Association of common polymorphisms with gestational diabetes mellitus in Japanese women: A case-control study. Endocr J.

[53] Kanthimathi S, Chidambaram M, Bodhini D, Liju S, Bhavatharini A, Uma R (2017). Association of recently identified type 2 diabetes gene variants with Gestational Diabetes in Asian Indian population. Mol Genet Genomics.

[54] Clausen TD, Mathiesen ER, Hansen T, Pedersen O, Jensen DM, Lauenborg J (2008). High prevalence of type 2 diabetes and pre-diabetes in adult offspring of women with gestational diabetes mellitus or type 1 diabetes the role of intrauterine hyperglycemia. Diabetes Care.

[55] Sandovici I, Smith NH, Nitert MD, Ackers-Johnson M, Uribe-Lewis S, Ito Y (2011). Maternal diet and aging alter the epigenetic control of a promoter-enhancer interaction at the Hnf4a gene in rat pancreatic islets. P Natl Acad Sci USA.

[56] Kim E, Kwak SH, Chung HR, Ohn JH, Bae JH, Choi SH (2017). DNA methylation profiles in sibling pairs discordant for intrauterine exposure to maternal gestational diabetes. Epigenetics-Us.

[57] Liu CT, Monda KL, Taylor KC, Lange L, Demerath EW, Palmas W (2013). Genome-wide association of body fat distribution in African ancestry populations suggests new loci. Plos Genet.

[58] Pan DN, Huang L, Zhu LHJ, Zou T, Ou JH, Zhou W (2015). Jmjd3-mediated H3K27me3 dynamics orchestrate brown fat development and regulate white fat plasticity. Dev Cell.

[59] Pramanik D, Campbell NR, Karikari C, Chivukula R, Kent OA, Mendell JT (2011). Restitution of tumor suppressor microRNAs using a systemic nanovector inhibits pancreatic cancer growth in mice. Mol Cancer Ther.

[60] Akao Y, Nakagawa Y, Hirata I, Iio A, Itoh T, Kojima K (2010). Role of anti-oncomirs miR-143 and-145 in human colorectal tumors. Cancer Gene Ther.

[61] Ibrahim AF, Weirauch U, Thomas M, Grunweller A, Hartmann RK, Aigner A (2011). MicroRNA replacement therapy for miR-145 and miR-33a is efficacious in a model of colon carcinoma. Cancer Research.

[62] Bauer J, Bastian BC (2006). Distinguishing melanocytic nevi from melanoma by DNA copy number changes: comparative genomic hybridization as a research and diagnostic tool. Dermatol Ther.

[63] Bastian BC, Olshen AB, LeBoit PE, Pinkel D (2003). Classifying melanocytic tumors based on DNA copy number changes. Am J Pathol.

[64] Bastian BC, LeBoit PE, Hamm H, Brocker EB, Pinkel D (1998). Chromosomal gains and losses in primary cutaneous melanomas detected by comparative genomic hybridization. Cancer Res.

[65] Namiki T, Yanagawa S, Izumo T, Ishikawa M, Tachibana M, Kawakami Y (2005). Genomic alterations in primary cutaneous melanomas detected by metaphase comparative genomic hybridization with laser capture or manual microdissection: 6p gains may predict poor outcome. Cancer Genet Cytogenet.

[66] Gerami P, Jewell SS, Morrison LE, Blondin B, Schulz J, Ruffalo T (2009). Fluorescence in situ hybridization (FISH) as an ancillary diagnostic tool in the diagnosis of melanoma. American Journal of Surgical Pathology.

[67] Morey AL, Murali R, McCarthy SW, Mann GJ, Scolyer RA (2009). Diagnosis of cutaneous melanocytic tumours by four-colour fluorescence in situ hybridisation. Pathology.

[68] Pouryazdanparast P, Newman M, Mafee M, Haghighat Z, Guitart J, Gerami P (2009). Distinguishing epithelioid blue nevus from blue nevus-like cutaneous melanoma metastasis using fluorescence in situ hybridization. Am J Surg Pathol.

[69] Gerami P, Wass A, Mafee M, Fang Y, Pulitzer MP, Busam KJ (2009). Fluorescence in situ hybridization for distinguishing nevoid melanomas from mitotically active nevi. Am J Surg Pathol.

[70] Abasolo A, Vargas MT, Rios-Martin JJ, Trigo I, Arjona A, Gonzalez-Campora R (2012). Application of fluorescence in situ hybridization as a diagnostic tool in melanocytic lesions, using paraffin wax-embedded tissues and imprint-cytology specimens. Clin Exp Dermatol.

[71] Martin V, Banfi S, Bordoni A, Leoni-Parvex S, Mazzucchelli L (2012). Presence of cytogenetic abnormalities in Spitz naevi: a diagnostic challenge for fluorescence in-situ hybridization analysis. Histopathology.

[72] Muhlbauer A, Momtahen S, Mihm MC, Wang J, Magro CM (2017). The correlation of the standard 5 probe FISH assay with melanocytic tumors of uncertain malignant potential. Ann Diagn Pathol.

[73] Zhou XR, Mao Y, Zhu JJ, Meng FY, Chen Q, Tao LH (2016). TGF-beta 1 promotes colorectal cancer immune escape by elevating B7-H3 and B7-H4 via the miR-155/miR-143 axis. Oncotarget.

[74] Yu B, Swatkoski S, Holly A, Lee LC, Giroux V, Lee CS (2015). Oncogenesis driven by the Ras/Raf pathway requires the SUMO E2 ligase Ubc9. P Natl Acad Sci USA.

[75] de Sio FRS, Barde I, Offner S, Kapopoulou A, Corsinotti A, Bojkowska K (2012). KAP1 regulates gene networks controlling T-cell development and responsiveness. Faseb J.

[76] Hatakeyama S (2017). TRIM family proteins: roles in autophagy, immunity, and carcinogenesis. Trends Biochem Sci.

[77] Xiao JQ, Lee ST, Xiao YY, Ma XM, Houseman EA, Hsu LI (2014). PTPRG inhibition by DNA methylation and cooperation with RAS gene activation in childhood acute lymphoblastic leukemia. Int J Cancer.

